# Let's go round again! Quality improvement through intentional rounding

**DOI:** 10.1186/cc12461

**Published:** 2013-03-19

**Authors:** P Doyle, F Cox, R Tollyfield, A Seraj

**Affiliations:** 1 Harefield Hospital, Harefield, UK

## Introduction

Harefield Hospital is a 150-bed cardiothoracic tertiary referral centre with transplantation, artificial heart, ECMO and primary angioplasty services. Our 35-bed critical care department consists of 18 intensive therapy unit, seven recovery and 10 high-dependency beds. Intentional rounds or proactive patient rounds were recognised by the Royal College of Physicians and the Royal College of Nursing [1] as structured, evidence-based processes for nurses to carry out regular checks with individual patients at set intervals. The senior nursing team decided to adapt this initiative to the intensive care setting in order to address clinical challenges and provide guidance for shift leaders to focus on key elements of care.

## Methods

Our intentional rounds, performed once per shift (twice daily), include two components. First, pressure area care - this component involves the shift leader checking whether key elements of pressure sore prevention have been performed. These include completion of the Waterlow risk assessment tool [2], noting the frequency of repositioning, use of lateral positioning and pressure-relieving pads. Second, renal replacement therapy rates - this element was identified as an area for focus after we established that our haemofiltration fluid use per hour of therapy was twice that of a near identical clinical setting. This pattern continued even after adopting similar therapy guidelines. The shift leader was guided to check whether therapy rates had been adjusted in line with latest biochemical results.

## Results

The incidence of pressure ulcers in the 4 months since the initiative began has averaged 2.25 per month compared with 7.8 per month prior to commencement of intentional rounding. Added to the rounding tool at the end of September 2012, RRT rates in the preceding 4 months averaged 31.5 ml/kg/hour over 24 hours, an 11.9% reduction from the previous average of 35.75 ml/kg/hour. If the pattern of RRT was to continue, this could equate to a cost saving of UK£40,000 per annum.

## Conclusion

The use of a modified targeted intentional rounding tool by the nursing shift leader can help ensure that best practice guidelines are adhered to. This strategy can improve patient outcomes and provide potentially significant fiscal benefits.

## References

[B1] RCP, RCN Ward Rounds in Medicine Principles for Best Practice 2012 London: Royal College of Physicians, Royal College of Nursing

[B2] Waterlow J The importance of accurate risk assessment and appropriate intervention in tissue viability Br J Nurs 2009 18 1090 19966723 10.12968/bjon.2009.18.18.44546

